# Macrophages on the run: Exercise balances macrophage polarization for improved health

**DOI:** 10.1016/j.molmet.2024.102058

**Published:** 2024-10-29

**Authors:** Yotam Voskoboynik, Andrew D. McCulloch, Debashis Sahoo

**Affiliations:** 1 Department of Bioinformatics and System Biology, Jacobs School of Engineering, University of California San Diego, San Diego, United States; 2 Department of Bioengineering, University of California San Diego, United States; 3 Department of Pediatrics, University of California San Diego, United States; 4 Department of Computer Science and Engineering, Jacob’s School of Engineering, University of California San Diego, United States; 5 Department of Medicine, University of California San Diego, United States

**Keywords:** Exercise biology, Macrophage polarization, Immune modulation, Tissue regeneration, Machine learning models, Muscle

## Abstract

**Objective:**

Exercise plays a crucial role in maintaining and improving human health. However, the precise molecular mechanisms that govern the body’s response to exercise or/compared to periods of inactivity remain elusive. Current evidence appears to suggest that exercise exerts a seemingly dual influence on macrophage polarization states, inducing both pro-immune response M1 activation and cell-repair-focused M2 activation. To reconcile this apparent paradox, we leveraged a comprehensive meta-analysis of 75 diverse exercise and immobilization published datasets (7000+ samples), encompassing various exercise modalities, sampling techniques, and species.

**Methods:**

75 exercise and immobilization expression datasets were identified and processed for analysis. The data was analyzed using boolean relationships which uses binary gene expression relationships in order to increase the signal to noise achieved from the data, allowing for the use of comparison across such a diverse set of datasets. We utilized a boolean relationship-aided macrophage gene model [1], to model the macrophage polarization state in pre and post exercise samples in both immediate exercise and long term training.

**Results:**

Our modeling uncovered a key temporal dynamic: exercise triggers an immediate M1 surge, while long term training transitions to sustained M2 activation. These patterns were consistent across different species (human vs mouse), sampling methods (blood vs muscle biopsy), and exercise type (resistance vs endurance), and routinely showed statistically significant results. Immobilization was shown to have the opposite effect of exercise by triggering an immediate M2 activation. Individual characteristics like gender, exercise intensity and age were found to impact the degree of polarization without changing the overall patterns. To model macrophages within the specific context of muscle tissue, we identified a focused gene set signature of muscle resident macrophage polarization, allowing for the precise measurement of macrophage activity in response to exercise within the muscle.

**Conclusions:**

These consistent patterns across all 75 examined studies suggest that the long term health benefits of exercise stem from its ability to orchestrate a balanced and temporally-regulated interplay between pro-immune response (M1) and reparative macrophage activity (M2). Similarly, it suggests that an imbalance between pro-immune and cell repair responses could facilitate disease development. Our findings shed light on the intricate molecular choreography behind exercise-induced health benefits with a particular insight on its effect on the macrophages within the muscle.

## Highlights

• Database of 75 exercise gene expression datasets, consisting of 7000+ samples.
• Exercise triggers an immediate M1 macrophage proinflammatory environment.
• Long term training leads to sustained M2 regenerative macrophage activation.
• Gender, age and exercise intensity affect the strength of macrophage activation.
• Identification of a gene model signature of muscle resident macrophage polarization.

## 1 Introduction

In 1902, Ralph Larrabee provided intriguing evidence suggesting parallels between the changes in white blood cell counts observed in Boston Marathon runners and those seen in specific disease states [2]. Notably he also noted a considerable leukocytosis of the inflammatory type, suggesting a potential link between extreme exercise and inflammatory responses. This early observation laid the groundwork for further investigations into the complex relationship between exercise intensity, immune system activation, and health outcomes, igniting an ongoing debate about the impact of exercise on the immune system.

Today we know exercise shows quantifiable and observable benefits to human health across multiple scales, but the specific genetic and biological processes and pathways underlying these benefits remain unclear [3]. This is primarily caused by individuals exhibiting significant physiological variations in their response to exercise training, coupled with the diverse methods, subjects and timelines used in studying this phenomenon, which impacts the potential for clear and reproducible analysis [[4], [5], [6]]. A deeper grasp of the metabolic and cellular impacts of exercise could lead to more targeted exercise approaches. Additionally, unraveling the molecular shifts induced by various exercise methods may hasten the identification of pharmaceutical targets for improving metabolic well-being. To combat the global pandemic of physical inactivity [7,8] and its associated toll of 5.3 million deaths annually, we must gain a better understanding of the fundamental principles governing physical activity’s benefits.

As the amount of gene expression data increases and more studies are conducted, accurate gene markers and gene signature models become important in allowing us to study specific cell types under conditions and experiments that did not originally aim to study those cell types. This also enables using such large amounts of data to draw consensus on the impact of specific extrinsic factors (such as exercise) on all aspects of the human body.

Macrophages, immune cells crucial in defending against infections and maintaining tissue balance and repair, exist in two distinct functional states; M1 which is associated with immune response and an inflammatory state, and M2 which is linked to an anti-inflammation tissue repair state [9]. Proper balance between these states occurs under normal *in vivo* circumstances [10]. Macrophages can adopt either the M1 or M2 phenotypes based on environmental cues and signals they receive. The M1/M2 classification is a simplified characterization of macrophage functions, best viewed as a spectrum rather than discrete states. Exercise has been shown to have pro-inflammatory effects [6,11], with several genes associated with M1 macrophages, such as IL-2 [12], IL-6 [13], IL-15 [14], and TNF1/TNF2 [15], upregulated post-exercise. However, exercise has also been demonstrated to expedite wound healing [[16], [17], [18]] and even prompt an increase in M2 macrophages [19]. The seemingly opposing response of Macrophage states from exercise creates confusion regarding the role of Macrophages in the long term benefits of exercise. In an effort to understand this phenomena, we compiled a meta-analysis of 75 previously published exercise-related datasets, encompassing Microarray, RNA-seq, and scRNA-Seq expression data from experiments involving short and long-term resistance, endurance, and immobilization training, sourced from both muscle biopsy and blood sampling, and spanning human, mice, and rat species[[20], [21], [22], [23], [24], [25], [26], [27], [28], [29], [30], [31], [32], [33], [34], [35], [36], [37], [38], [39], [40], [41], [42], [43], [44], [45], [46], [47], [48], [49], [50], [51], [52], [53], [54], [55], [56], [57], [58], [59], [60], [61], [62], [63], [64], [65], [66], [67], [68], [69], [70], [71], [72], [73], [74], [75], [76], [77], [78], [79], [80], [81], [82], [83], [84], [85], [86], [87], [88], [89], [90], [91], [92], [93], [94]].

**Figure 1:**
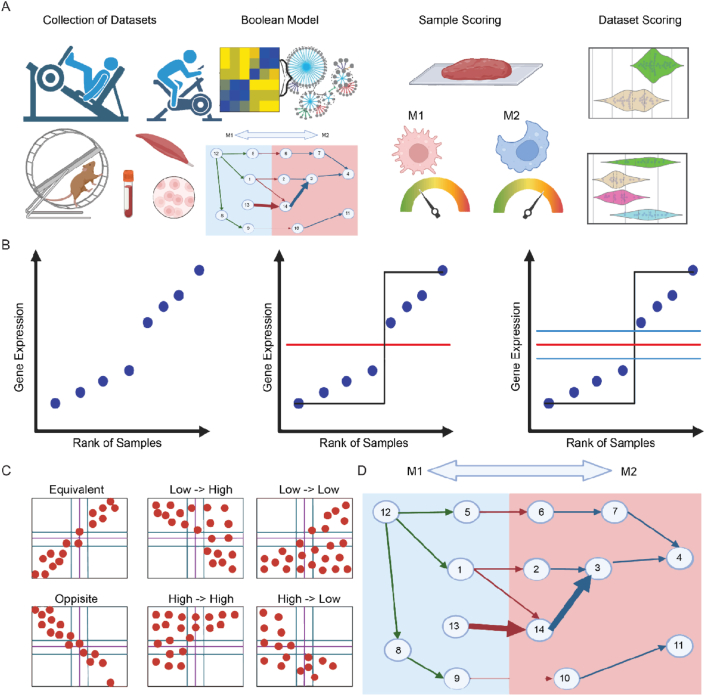
**Boolean workflow for analyzing macrophage polarization changes in exercise datasets.** A) Project Outline, with initial selection of datasets for meta-analysis, applying the macrophage polarization boolean model to each dataset to calculate a “macrophage score” for each sample, with lower scores representing M1 activation and higher scores representing M2 activation, and finally the comparing of the “macrophage scores” in pre and post exercise samples to measure the effect of exercise on macrophage polarization. B) StepMiner algorithm, in which samples are ranked by the expression of the selected genes, and a step function is fitted to the ranked data to minimize the sum of square error of the fitted data. The two sides of this threshold and margin allow for the binarization of the gene’s expression into “low” and “high” values. C) Boolean relationship scatter plots of the six possible boolean relationships between two genes. A Boolean implication relationship is identified if any of the scatter plot quadrants, which are split based on each gene’s StepMiner threshold, or two diagonally opposite ones, are sparsely populated. D) Formation of Boolean network. Using equivalent gene clusters as nodes and the boolean relationships between the clusters as the edges and network can be created from the boolean relationships. This network can then be oriented by condition and using machine learning to find a path from one condition to the other that can accurately separate between samples from each condition.

Identifying patterns within these datasets proves challenging due to their inherent diversity. To overcome this obstacle, we employed Boolean logic, a powerful mathematical method that simplifies gene expression relationships into binary terms (high/low, 1/0, or positive/negative). This method increases the signal to noise ratio from diverse datasets and allows for the identification of relationships between genes that consistently appears in almost all contexts [95], and which would be missed by simple differential expression analysis which focuses solely on individual gene counts. We employed a Boolean-assisted Macrophage Polarization model [1] on 75 existing exercise-related datasets (Figure 1A), to reveal common and consistent elements of both the immediate and long term exercise training’s effect on Macrophage states. And further identified a subset of 25 (3 M1, 22 M2) genes that can accurately predict pre vs post exercise and trained vs control within muscle tissue samples. Our findings unequivocally demonstrate that immediate exercise triggers the activation of M1 macrophages, while long term exercise yields the inverse effect of M2 activation.

## 2 Methods

### 2.1 Compiling and annotating a comprehensive database of existing exercise datasets

The network meta-analysis was performed according to the Preferred Reporting Items for Systematic Reviews and Meta-Analyses (PRISMA) extension of the Network Meta-check Analysis List [96]. Electronic searches for the terms: exercise or training, alongside either aerobic endurance resistance or strength (leading to 8 search terms), as well as immobilization and inactivity exercise were performed across four electronic databases, viz. PubMed, Embase, Web of Science, and the Cochrane Library, from inception until July 2024. The found papers were then cross referenced with genomic expression datasets from the Gene Expression Omnibus (GEO) expression databases [97] and filtered based the following criteria: 1) It must contain gene expression count data (RNA-Seq, Microarray, scRNA-Seq). 2) There are at least two sampling timepoints/groups, encompassing a pre-exercise/untrained condition and a post-exercise/trained condition. The specific duration post-exercise or any additional experimental condition groups were not considered in the inclusion process. Muscle immobilization studies with a minimum of two experimental groups were also included in the analysis but were kept distinct from the rest of the datasets. 3) The dataset contains at least 10 samples of either exercise or control. 4) The sample species must be mammalian. 5) Each species needed to have at least two distinct datasets for inclusion. 6) The dataset must be associated with a corresponding peer reviewed published paper.

Additionally, 3 other non-exercise datasets (GSE142068, GSE230102, GSE59927) [[98], [99], [100]] which contain spleen and heart or skeletal muscle tissue data, and one pooled dataset of 170 published macrophage samples (GSE134312) [101] were processed for the comparison with the muscle tissue specific signature.

### 2.2 Data collection and annotation

Publicly available microarray, RNA-Seq, and scRNA-Seq databases were downloaded from the National Center for Biotechnology Information (NCBI) Gene Expression Omnibus (GEO) website. Gene expression summarization was performed by normalizing Affymetrix platforms by RMA (Robust Multichip Average) [102] and RNASeq platforms by computing CPM (Counts Per Million) [103] values whenever normalized data were not available in GEO. We used log2(CPM + 1) as the final gene expression value for analyses. We also used public data normalized with Reads Per Kilobase per Million mapped reads (RPKM) with final gene expression values of log2(RPKM + 1) [103].

### 2.3 StepMiner analysis

StepMiner is a computational tool designed for detecting incremental transitions within time-series data [104]. Here we used this method as previously described in Sahoo 2007 [104]. Illustrated in Figure 1B StepMiner is facilitated through an adaptive regression approach that identifies the optimal step, based on the sum-of-square errors. The steps are positioned between time points that exhibit the most pronounced shift from low to high expression levels. In order to fit a step function, the algorithm assesses all potential step positions. For each position, it calculates the average of values on both sides of the step for the constant segments. The algorithm selects step positions that minimize the square error with the fitted data. Finally, a regression test statistic is computed as follows:

$$F\;stat=\frac{\sum_{i=1}^{n}{\left(\hat{X_{i}}-\hat{X}\right)}^{2}/m-1\;}{\sum_{i=1}^{n}{X_{i}-\hat{X_{i}}}^{2}/n-m}$$

where $X_{i}$ for i=1 to n are the values, $\hat{X_{i}}$ for i=1 to n are fitted values. m is the degrees of freedom used for the adaptive regression analysis. $\hat{X}$ is the average of all the values: $X=\frac{1}{n}*\sum_{j=1}^{n}X_{j}$. For a step position at k, the fitted values $\hat{X_{l}}$ are computed by using $\frac{1}{k}*\sum_{j=1}^{n}X_{j}$ for i=1 to k and $\frac{1}{\left(n-k\right)}*\sum_{j=k+1}^{n}X_{j}$ for i=k+1.

The StepMiner algorithm operates on gene expression levels, transforming them into Boolean values (high and low). In this process, expression values are initially sorted from low to high, and a rising step function is fitted to the series to identify a threshold. The middle of the step is designated as the StepMiner threshold. A noise margin of a 2-fold change is applied around the threshold to account for intermediate values, which are disregarded during Boolean analysis.

### 2.4 Boolean analysis

Boolean logic is a fundamental mathematical relationship between two values, such as high/low, 1/0, or positive/negative [105]. When applied to gene expression data, Boolean analysis involves converting expression levels into two distinct values. In a scatter plot, four potential quadrants are determined based on Boolean values: (low, low), (low, high), (high, low), and (high, high) [95]. A Boolean implication relationship is identified if any of these quadrants, or two diagonally opposite ones, are sparsely populated. According to this criterion, there are six types of Boolean implication relationships. Figure 1C shows these six possible relationships, with two of them being symmetric relationships in which there are two sparse quadrants: equivalent (corresponding to positively correlated genes) and opposite (corresponding to highly negatively correlated genes). The other four Boolean relationships are asymmetric, each corresponding to one sparsely populated quadrant: (low => low), (high => low), (low => high), and (high => high). BooleanNet statistics are utilized to assess quadrant sparsity and the significance of Boolean implication relationships. When considering a gene pair, A and B, four quadrants are identified using StepMiner thresholds on A and B, while disregarding intermediate values defined by a noise margin of a 2-fold change (±0.5 around the StepMiner threshold). The number of samples in each quadrant, designated as a00, a01, a10, and a11, are distinct from the variable X used in the previous equation for F stat. The total number of samples where gene expression values for A and B are low is calculated using the following equations:

$$nA_{low}=\left(a_{00}+a_{01}\right),nB_{low}=\left(a_{00}+a_{10}\right)$$

The total number of samples considered is computed using the following equation.

$$total=a_{00}+a_{01}+a_{10}+a_{11}$$

The expected number of samples in each quadrant is computed by assuming independence between A and B. The following equation is used to compute the expected number of samples.

$$n=a_{ij},\hat{n}=\left(nA_{low}/total*nB_{low}/total\right)*total$$

To check whether a quadrant is sparse, a statistical test for $\left(e_{00}>a_{00}\right)$ or ($\hat{n}>n$) is performed by computing $S_{00}$ and $p_{00}$ using the following equations. A quadrant is considered sparse if $S_{00}$ is high ($\hat{n}>n)$ and $p_{00}$ is small.

$$S_{ij}=\frac{\hat{n}-n}{\sqrt{\hat{n}}}$$

$$p_{00}=\frac{1}{2}\left(\frac{a_{00}}{\left(a_{00}+a_{01}\right)}+\frac{a_{00}}{\left(a_{00}+a_{10}\right)}\right)$$

A suitable threshold is chosen for $S_{00}$ > sThr and $p_{00}$ < pThr to check sparse quadrant. A Boolean implication relationship is identified when a sparse quadrant is discovered using the following equation.

$$Boolean\;Implication=\left(S_{ij}>sThr,p_{ij}<pThr\right)$$

A relationship is called Boolean equivalent if top-left and bottom-right quadrants are sparse.

$$Equivalent=\left(S_{01}>sThr,P_{01}<pThr,S_{10}>sThr,P_{10}<pThr\right)$$

Boolean opposite relationships have sparse top-right ($a_{11}$) and bottom-left ($a_{00}$) quadrants.

$$Opposite=\left(S_{00}>sThr,P_{00}<pThr,S_{11}>sThr,P_{11}<pThr\right)$$

Boolean equivalent and opposite are symmetric relationships because the relationship from A to B is the same as from B to A. Asymmetric relationship forms when there is only one quadrant sparse (A low => B low: top-left; A low => B high: bottom-left; A high => B high: bottom-right; A high => B low: top-right). These relationships are asymmetric because the relationship from A to B is different from B to A.

A low => B high is discovered if the bottom-left ($a_{00}$) quadrant is sparse and this relationship satisfies following conditions.

$$A\;low=>\;B\;high=\left(S_{00}>sThr,P_{00}<pThr\right)$$

Similarly, A low => B low is identified if the top-left ($a_{01}$) quadrant is sparse.

$$A\;low=>\;B\;low=\left(S_{01}>sThr,P_{01}<pThr\right)$$

A high => B high Boolean implication is established if the bottom-right ($a_{10}$) quadrant is sparse as described below.

$$A\;high=>\;B\;high=\left(S_{10}>sThr,P_{10}<pThr\right)$$

Boolean implication A high => B low is found if the top-right ($a_{11}$) quadrant is sparse using the following equation.

$$A\;high=>\;B\;low=\left(S_{11}>sThr,P_{11}<pThr\right)$$

For each quadrant a statistic $S_{ij}$ and an error rate $p_{ij}$ is computed. $S_{ij}>sThr$ and $p_{ij}<pThr$ are the thresholds used on the BooleanNet statistics to identify Boolean implication relationships.

### 2.5 Macrophage polarization score

The “macrophage score” was calculated using the boolean macrophage polarization previously described in Ghosh et al., 2023 [1]. In short, a boolean network was created to differentiate between M1 and M2 macrophage samples. As shown in Figure 1D genes with the equivalent boolean relationship are clustered together, with single gene clusters being pruned out. A network can then be created, with the gene clusters as nodes and the boolean relationships between the clusters as the edges. This network can then be oriented by the condition and using machine learning and artificial intelligence to find a path from one condition to the other (in this case M1 and M2 macrophages) that can accurately separate between samples from each condition. The macrophage polarization model used Boolean relationships as a a basis to create a path of 338 genes which accurately separated M1 vs M2 macrophages in both training and validation independent clinically prescribed macrophage datasets (M1 = 48 genes, M2 = 290 genes). In Figure 3 the scores for GSE27536 and GSE14798 were calculated with only the M1 associated genes due to the disease status of all the samples in those datasets. A combined score was calculated for a specified boolean pathway. To derive this score, the genes within each cluster were initially normalized and then averaged. The normalization of gene expression values followed a modified Z-score approach, centered around the StepMiner threshold (formula = (expr – (SThr + 0.5)))/3∗stdev). A weighted linear combination of the cluster averages along the boolean pathway was employed to generate a score for each sample. Subsequently, the samples were organized based on the final weighted scores and the linearly combined score. The direction of the pathway was determined by the connection from a M1 cluster to a M2 cluster, A noise margin for this composite score was computed, adhering to the same linearly weighted combined score with a 2-fold change allowance (±0.5 around the StepMiner threshold).

### 2.6 Identification of a new signature of muscle resident macrophage polarization

Genes were taken from the original macrophage model [1], and tested on a single muscle exercise dataset [68], with the genes associated weight being assigned based on its original cluster. Genes which were able to significantly separate between the pre vs post exercise samples with an accuracy of absolute value |0.7| ROC-AUC were included in the identified gene set model groups. All genes with ROC-AUC value above 0.5 were placed into Group 1 while all genes with a ROC-AUC below 0.5 were placed into Group 2. The new gene set model groups M1-M2 prediction was validated with a pooled dataset of 170 published macrophage samples (GSE134312) [101], while the exercise prediction was validated on all 20 of the muscle tissue datasets from the 75 assembled database. The two resulting gene set model groups were further compared between spleen and heart/skeletal muscle tissues in 3 non-exercise datasets (GSE142068, GSE230102, GSE59927) [[98], [99], [100]] and 1 exercise (GSE224146) dataset [106]. Because the aim was to identify the macrophage signature within the compared tissues (spleen and heart/skeletal muscle), the tissue comparison was done with all genes given an equal weight of +1.

### 2.7 Statistical analyses

The gene signature is utilized for categorizing sample groups, and the effectiveness of the multi-class classification is assessed through ROC-AUC (Receiver Operating Characteristics Area Under The Curve) values. Standard t-tests were carried out using the python scipy.stats.ttest_ind package (version 1.11.3) with parameters for Welch’s Two Sample t-test (unpaired, unequal variance, and unequal sample size; equal_var = False).

### 2.8 Missing gene data

For each dataset in our database, whenever expression data for a gene was missing for one or more samples it was left blank and ignored in the Boolean relationship calculations. If the gene with missing data was within either the macrophage model and Signature of Muscle Resident Macrophage Polarization genes sets it was not included in the composite score calculations. Because the boolean networks are built on clusters of equivalent genes, which will have highly similar expression across all the genes in the same cluster, it is the expression levels of the whole cluster that is important in the macrophage and muscle resident composite “sample scores”. As such the scores were calculated from all of the cluster genes which were present within each particular dataset, ignoring any model genes which did not appear in that dataset. In the case of microarray datasets, in which often multiple probes align to the same gene, all instances of aligned probes were used in the sample score calculations.

## 3 Results

### 3.1 Compiling a comprehensive database of existing exercise datasets

**Figure 2:**
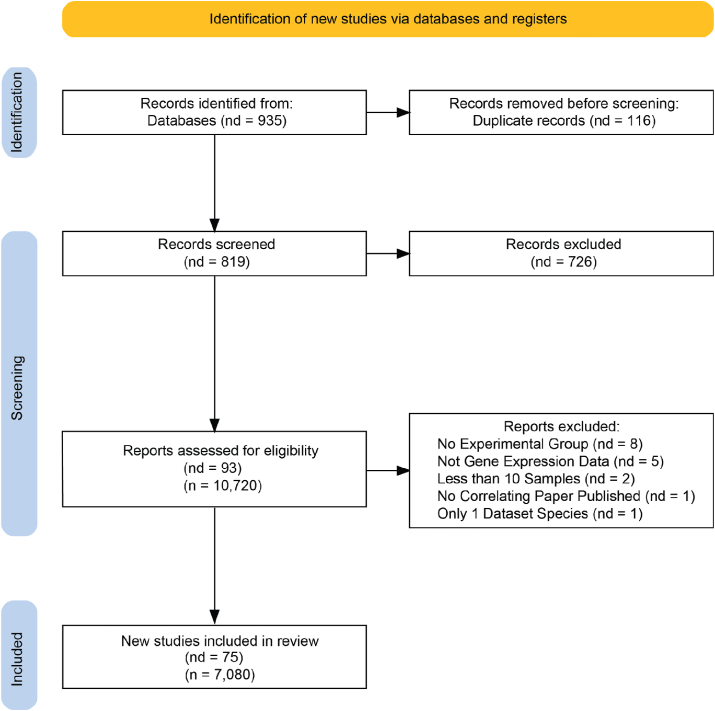
**PRISMA flowchart showing the selection of datasets for the meta-analysis**, with initial screening of the terms: exercise or training, alongside either aerobic endurance resistance or strength across PubMed, Embase, Web of Science, and the Cochrane Library leading to 819 non-duplicate studies. Number of databases is listed (nb) alongside the total number of samples (n). Of these 93 were retrieved for full text, 8 studies were filtered for containing only control non-exercised subjects, 5 were removed for not containing gene expression data, 3 for having no correlated published paper and 2 for belonging to a species for which no other dataset could be found.

We screened and analyzed published exercise datasets to build a comprehensive gene expression database. Four electronic databases, PubMed, Embase, Web of Science, and the Cochrane Library, were searched from inception to July 2024 for the terms: exercise or training, alongside either aerobic endurance resistance or strength (leading to 8 search terms), as well as immobilization and inactivity exercise. Genomic expression datasets from the Gene Expression Omnibus (GEO) expression databases [97] were cross-referenced with the search results. Datasets were selected based on the following criteria: 1) The dataset must contain gene expression count data (e.g., RNA-Seq, Microarray, scRNA-Seq). 2) The dataset must have at least two sampling timepoints/groups, encompassing a pre-exercise/untrained condition and a post-exercise/trained condition. The specific duration post-exercise or any additional experimental condition groups were not considered in the inclusion process. Muscle immobilization studies with a minimum of two experimental groups were also included in the analysis but were kept distinct from the rest of the datasets. 3) The dataset contains at least 10 samples of either exercise or control. 4) The samples are from mammalian species. 5) Each species must have at least two distinct datasets. 6) The dataset must be associated with a corresponding peer-reviewed published paper. After eliminating duplicates, we initially retrieved a total of 935 records from the databases. Following the screening of titles and abstracts, we thoroughly reviewed 93 potentially eligible reports in full text. Ultimately, 75 studies met the inclusion criteria, while 18 were excluded [[20], [21], [22], [23], [24], [25], [26], [27], [28], [29], [30], [31], [32], [33], [34], [35], [36], [37], [38], [39], [40], [41], [42], [43], [44], [45], [46], [47], [48], [49], [50],[51], [52], [53], [54], [55], [56], [57], [58], [59], [60], [61], [62], [63], [64], [65], [66], [67], [68], [69], [70], [71], [72], [73], [74], [75], [76], [77], [78], [79], [80], [81], [82], [83], [84], [85], [86], [87], [88], [89], [90], [91], [92],^107^,^108^]. Figure 2 illustrates the Preferred Reporting Items for Systematic reviews and Meta-Analyses (PRISMA) process for selecting eligible studies and outlines the reasons for exclusion. Table 1 shows each of the datasets included in the analysis, alongside relevant information of Species, Experiment and Exercise Types, as well as number of Samples and number of samples in different subgroups (Gender, Tissue Type, Age, etc.) and the relevant associated paper.

**Table 1:** **Dataset information table**, containing each of the 75 public datasets used in this analysis. Each row relates to a single dataset with the columns containing information of: the gene expression omnibus GSE id #, the total number of samples in the dataset, the species from which the dataset was collected, the sequencing type of the data (microarray rna-seq single-cell rna, etc.), the exercise time in which the data exists (immediate within 6 h post exercise, or long term training of at least 1 month), as well as breakdowns of the number of samples within different tissue types, genders and age groups, the final column is the relevant paper associated with the data itself.

ID	Number Samples	Species	Experiment Type	Tissues	Genders	Ages	Citation
E-MEXP-740	32	Homo sapiens	Transcription profiling by array	{}	{‘Male’: 32}	{‘69 to 73’: 32}	https://doi.org/10.1093/gerona/62.10.1088
E-MTAB-1788	1655	Homo sapiens	Transcription profiling by array	{}	{‘Unknown’: 45, ‘mixed’: 428, ‘female’: 257, ‘male’: 372}	{‘2’: 26, ‘0.8’: 28, ‘1.8’: 16, ‘ ’: 1032}	https://doi.org/10.1186/s13395-015-0059-1
GSE104079	58	Mus musculus	Expression profiling by array	{‘Soleus’: 27, ‘Liver’: 31}	{‘Male’: 58}	{‘6 month old’: 58}	https://doi.org/10.1096/fj.201701378RR
GSE104999	24	Homo sapiens	Expression profiling by array	{‘Vastus’: 24}	{‘Male’: 24}	{}	https://doi.org/10.1152/ajpregu.00452.2017
GSE109657	22	Homo sapiens	Expression profiling by array	{‘Vastus’: 22}	{‘Male’: 22}	{}	https://doi.org/10.1038/s41598-018-35115-x
GSE110747	44	Mus musculus	Expression profiling by array	{‘Liver’: 44}	{‘Male’: 44}	{‘11–12 weeks’: 44}	https://doi.org/10.3390/nu10050547
GSE111555	142	Homo sapiens	Expression profiling by array	{‘Total’: 84, ‘Peripheral’: 32, ‘Vastus’: 26}	{‘Male’: 142}	{‘30’: 20, ‘--’: 2, ‘28’: 26, ‘22’: 10, ‘29’: 18, ‘23’: 6, ‘19’: 12, ‘21’: 10, ‘25’: 10, ‘27’: 24, ‘26’: 4}	https://doi.org/10.1002/lipd.12155
GSE117070	82	Homo sapiens	Expression profiling by array	{}	{}	{}	https://doi.org/10.1016/j.celrep.2017.10.040
GSE117525	259	Homo sapiens	Expression profiling by array	{‘Vastus’: 259}	{‘Female’: 81, ‘male’: 178}	{18: 7, 19: 7, 20: 5, 21: 12, 22: 7, 23: 10, 25: 2, 26: 1, 27: 1, 29: 1, 65: 11, 66: 20, 67: 18, 68: 14, 69: 8, 70: 11, 71: 7, 72: 13, 73: 10, 74: 12, 75: 3, 76: 16, 77: 8, 78: 4, 79: 5, 80: 6, 81: 6, 82: 1, 83: 3, 84: 9, 85: 5, 86: 4, 87: 2, 89: 3, 90: 1, 91: 3, 93: 1, 95: 1, 96: 1}	https://doi.org/10.1002/jcsm.12099
GSE11803	12	Mus musculus	Expression profiling by array	{}	{}	{}	https://doi.org/10.1016/j.cell.2008.06.051
GSE120862	84	Homo sapiens	Expression profiling by high throughput sequencing	{‘Skeletal’: 84}	{‘Male’: 84}	{33: 12, 29: 12, 21: 48, 22: 12}	https://doi.org/10.1152/ajpendo.00449.2018
GSE122671	88	Homo sapiens	Expression profiling by array	{}	{‘Male’: 88}	{}	https://doi.org/10.1016/j.jff.2019.04.022
GSE126001	16	Mus musculus	Expression profiling by high throughput sequencing	{‘Gastrocnemius’: 16}	{‘Male’: 16}	{‘20-weeks old’: 16}	https://doi.org/10.1126/science.aat3987
GSE126296	28	Homo sapiens	Expression profiling by array	{‘Skeletal’: 28}	{‘Female’: 7, ‘male’: 7}	{33: 2, 21: 2, 22: 2, 23: 2, 24: 8, 25: 2, 26: 2, 27: 4, 29: 2, 30: 2}	https://doi.org/10.1371/journal.pone.0223024
GSE139258	24	Homo sapiens	Expression profiling by array	{‘Vastus’: 24}	{‘Male’: 11, ‘female’: 13}	{}	https://doi.org/10.14814/phy2.14416
GSE140089	41	Homo sapiens	Expression profiling by high throughput sequencing	{‘Skeletal’: 41}	{‘Male’: 31, ‘female’: 10}	{23: 1, 24: 1, 25: 1, 27: 1, 30: 2, 32: 2, 33: 1, 34: 1, 35: 1, 37: 1, 50: 1, 54: 2, 58: 1, 60: 1, 62: 1, 63: 1, 65: 2, 66: 3, 67: 3, 69: 2, 71: 4, 73: 2, 74: 1, 75: 2, 76: 2, 79: 1}	https://doi.org/10.3389/fphys.2020.00653
GSE144304	80	Homo sapiens	Expression profiling by high throughput sequencing	{‘Vastus’: 80}	{‘Male’: 41, ‘female’: 39}	{26.74: 1, 77.6: 1, 77.88: 1, 77.97: 1, 77.77: 1, 77.42: 1, 78.29: 1, 78.92: 1, 78.38: 1, 78.07: 1, 27.4: 1, 79.81: 1, 79.03: 1, 80.32: 1, 80.79: 1, 80.68: 1, 20.83: 1, 22.75: 1, 81.19: 1, 81.27: 1, 81.17: 1, 81.95: 1, 76.52: 1, 82.77: 2, 82.4: 1, 76.83: 1, 82.7: 1, 24.01: 1, 83.93: 1, 83.03: 1, 75.97: 1, 83.72: 1, 25.02: 1, 24.54: 1, 75.23: 1, 75.75: 1, 75.9: 1, 77.03: 1, 77.4: 1, 78.7: 1, 77.59: 1, 77.05: 1, 77.54: 2, 80.27: 1, 81.14: 1, 82.44: 1, 83.16: 1, 81.1: 1, 81.65: 1, 82.16: 1, 90.07: 1, 86.61: 1, 86.15: 1, 84.0: 1, 85.12: 1, 24.5: 1, 75.13: 1, 75.31: 1, 20.47: 1, 20.63: 1, 20.39: 1, 20.64: 1, 20.81: 1, 21.73: 1, 21.25: 1, 21.46: 1, 21.42: 1, 22.49: 1, 22.7: 1, 23.35: 1, 23.73: 1, 23.96: 1, 23.82: 1, 23.51: 1, 24.14: 1, 75.95: 1, 25.28: 1, 76.55: 1}	https://doi.org/10.1007/s11357-023-00750-4
GSE155271	13	Homo sapiens	Expression profiling by array	{‘Vastus’: 13}	{‘Male’: 13}	{}	https://doi.org/10.3390/jcm9123951
GSE155933	230	Homo sapiens	Expression profiling by array	{‘Vastus’: 230}	{}	{‘PRE young’: 90, ‘POST young’: 103, ‘PRE middle’: 19, ‘POST middle’: 18}	https://doi.org/10.1016/j.celrep.2020.107980
GSE165630	14	Homo sapiens	Expression profiling by high throughput sequencing	{}	{‘Male’: 14}	{0.0: 5, 4.5: 1, 6.9: 1, 7.0: 1, 8.0: 1, 9.0: 2, 6.0: 1, 14.0: 1, 16.0: 1}	https://doi.org/10.3390/ijms22041539
GSE16907	60	Homo sapiens	Expression profiling by array	{‘M.’: 60}	{‘Female’: 60}	{‘Postmenopausal’: 60}	https://doi.org/10.1007/s11357-010-9140-1
GSE17190	24	Rattus norvegicus	Expression profiling by array	{‘Musculus’: 24}	{‘Female’: 24}	{‘8 months’: 24}	https://doi.org/10.1096/fj.10-157313
GSE178262	40	Mus musculus	Expression profiling by high throughput sequencing	{‘Skeletal’: 40}	{}	{}	https://doi.org/10.1113/JP281535
GSE1786	24	Homo sapiens	Expression profiling by array	{}	{}	{65: 2, 66: 2, 67: 2, 68: 2, 71: 2, 72: 2, 76: 6, 80: 2, 56: 2, 63: 2}	https://doi.org/10.1249/01.mss.0000181838.96815.4d
GSE179394	138	Mus musculus	Expression profiling by high throughput sequencing	{}	{}	{}	https://doi.org/10.1016/j.metabol.2021.154873
GSE1832	15	Homo sapiens	Expression profiling by array	{}	{}	{}	https://doi.org/10.1186/gb-2003-4-10-r61
GSE19062	64	Homo sapiens	Expression profiling by array	{‘Skeletal’: 64}	{‘Male’: 64}	{18: 8, 19: 12, 20: 16, 21: 4, 22: 8, 23: 8, 25: 4, 26: 4}	https://doi.org/10.1371/journal.pone.0010695
GSE19420	42	Homo sapiens	Expression profiling by array	{}	{}	{64: 3, 66: 4, 67: 3, 68: 1, 48: 3, 49: 4, 51: 2, 54: 3, 56: 4, 57: 3, 58: 2, 59: 2, 60: 3, 61: 3, 62: 2}	https://doi.org/10.1210/jc.2011-3454
GSE198266	24	Mus musculus	Expression profiling by high throughput sequencing	{‘Soleus’: 12, ‘Gastrocnemius’: 12}	{‘Male’: 24}	{‘84-weeks-old’: 12, ‘9-weeks-old’: 12}	https://doi.org/10.18632/aging.204024
GSE199225	61	Homo sapiens	Expression profiling by high throughput sequencing	{}	{‘Female’: 61}	{38: 6, 20: 12, 23: 6, 25: 18, 28: 1, 30: 18}	https://doi.org/10.1113/JP282954
GSE21496	21	Homo sapiens	Expression profiling by array	{‘Vastus’: 21}	{}	{}	https://doi.org/10.1152/japplphysiol.00444.2010
GSE221210	140	Mus musculus	Expression profiling by high throughput sequencing	{‘Skeletal’: 140}	{}	{}	https://doi.org/10.1038/s42255-023-00891-y
GSE230102	499	Mus musculus	Expression profiling by high throughput sequencing	{‘Heart’: 100, ‘Gonadal’: 100, ‘Liver’: 100, ‘quadriceps’: 99, ‘Brown’: 100}	{‘Female’: 499}	{}	https://doi.org/10.1016/j.celrep.2023.112499
GSE236600	99	Homo sapiens	Expression profiling by high throughput sequencing	{‘’: 18, ‘Vastus’: 81}	{‘’: 18, ‘male’: 81}	{‘ OH’: 18, ‘old’: 18, ‘ LLE’: 32, ‘ YE’: 31}	https://doi.org/10.1038/s41598-019-57110-6
GSE23697	70	Homo sapiens	Expression profiling by array	{‘Vastus’: 70}	{‘Male’: 70}	{18: 10, 19: 10, 20: 18, 21: 18, 22: 4, 25: 2, 26: 4, 27: 2, 30: 2}	https://doi.org/10.1096/fj.10-177105
GSE24235	28	Homo sapiens	Expression profiling by array	{}	{‘Male’: 6, ‘female’: 4}	{‘Young’: 28}	https://doi.org/10.1186/1471-2164-11-659
GSE242354	915	Rattus norvegicus	Expression profiling by high throughput sequencing	{‘Liver’: 54, ‘Vastus’: 50, ‘spleen’: 50, ‘Hippocampus’: 56, ‘testes’: 25, ‘Brown’: 52, ‘Adrenal’: 52, ‘Ovaries’: 24, ‘Vena’: 30, ‘Kidney’: 52, ‘Gastrocnemius’: 62, ‘white’: 52, ‘heart’: 52, ‘Lung’: 52, ‘Paxgene’: 52, ‘Colon’: 50, ‘Hypothalamus’: 50, ‘small’: 50, ‘Cortex’: 50}	{}	{}	https://doi.org/10.1016/j.xgen.2023.100421
GSE250122	48	Homo sapiens	Expression profiling by array	{}	{}	{}	https://doi.org/10.3390/ijms25052881
GSE252357	61	Homo sapiens	Expression profiling by high throughput sequencing	{‘Skeletal_muscle’: 61}	{‘Male’: 38, ‘female’: 23}	{33: 5, 34: 5, 35: 10, 37: 5, 38: 5, 25: 5, 26: 5, 27: 3, 28: 5, 29: 9, 30: 4}	https://doi.org/10.1101/2024.03.26.586857
GSE27285	39	Homo sapiens	Expression profiling by array	{‘Vastus’: 39}	{}	{}	https://doi.org/10.1152/physiolgenomics.00073.2011
GSE27536	54	Homo sapiens	Expression profiling by array	{‘Musculus’: 54}	{}	{}	https://doi.org/10.1371/journal.pcbi.1002129
GSE28422	110	Homo sapiens	Expression profiling by array	{‘Vastus’: 110}	{‘Male’: 56, ‘female’: 54}	{‘Young’: 62, ‘old’: 48}	https://doi.org/10.1152/japplphysiol.00435.2011
GSE28498	52	Homo sapiens	Expression profiling by array	{‘Blood’: 52}	{‘Male’: 52}	{16: 2, 17: 2, 18: 4, 19: 4, 20: 14, 21: 8, 22: 8, 23: 2, 24: 4, 30: 4}	https://doi.org/10.1007/s00421-011-2048-3
GSE28998	14	Homo sapiens	Expression profiling by array	{‘Biceps’: 14}	{‘Female’: 6, ‘male’: 8}	{}	https://doi.org/10.1152/japplphysiol.00860.2011
GSE33603	55	Homo sapiens	Expression profiling by array	{‘Vastus’: 55}	{}	{‘L’: 25, ‘R’: 30}	https://doi.org/10.1126/scitranslmed.3002882
GSE33886	14	Homo sapiens	Expression profiling by array	{}	{}	{}	https://doi.org/10.1152/ajpendo.00356.2012
GSE34788	120	Homo sapiens	Expression profiling by array	{}	{‘Female’: 120}	{}	https://doi.org/10.1111/ahg.12006
GSE3606	20	Homo sapiens	Expression profiling by array	{}	{}	{}	https://doi.org/10.1152/japplphysiol.00066.2006
GSE40551	16	Homo sapiens	Expression profiling by array	{‘Skeletal’: 16}	{‘Male’: 16}	{}	https://doi.org/10.1172/JCI64526
GSE41769	36	Homo sapiens	Expression profiling by array	{‘Vastus’: 36}	{‘Male’: 36}	{‘53 years’: 8, ‘44 years’: 8, ‘55 years’: 4, ‘56 years’: 12, ‘52 years’: 4}	https://doi.org/10.1371/journal.pone.0051066
GSE4252	15	Homo sapiens	Expression profiling by array	{}	{}	{}	https://doi.org/10.1096/fj.04-3149fje
GSE43219	28	Homo sapiens	Expression profiling by array	{‘Skeletal’: 28}	{‘Female’: 5, ‘male’: 9}	{}	https://doi.org/10.1371/journal.pone.0127089
GSE43760	24	Homo sapiens	Expression profiling by array	{‘Vastus’: 24}	{‘Female’: 24}	{}	https://doi.org/10.1113/expphysiol.2013.072710
GSE43856	64	Homo sapiens	Expression profiling by array	{‘Blood’: 8, ‘skeletal’: 8}	{‘Male’: 64}	{}	https://doi.org/10.1152/japplphysiol.00143.2013
GSE44051	24	Homo sapiens	Expression profiling by array	{‘Primary’: 24}	{‘Female’: 6, ‘male’: 18}	{37: 2, 19: 2, 22: 4, 24: 2, 25: 6, 26: 4, 27: 2, 29: 2}	https://doi.org/10.1152/ajpcell.00043.2013
GSE46075	56	Homo sapiens	Expression profiling by array	{}	{‘Male’: 56}	{‘After running at 80% VO2 peak for 30 min on a treadmill’: 8, ‘after 30 min of recovery period (MT)’: 8, ‘after 60 min of recovery period (MT)’: 8, ‘after ramp test to exaustion’: 8, ‘after 30 min of recovery period (RTE)’: 8, ‘before exercise (1 week later)’: 8, ‘before exercise’: 8}	https://doi.org/10.1186/1472-6793-13-9
GSE467	36	Rattus norvegicus	Expression profiling by array	{}	{}	{}	https://doi.org/10.1113/jphysiol.2002.021220
GSE51216	32	Homo sapiens	Expression profiling by array	{‘Whole’: 32}	{‘Male’: 32}	{49: 1, 50: 4, 52: 8, 53: 4, 54: 1, 55: 1, 57: 4, 60: 6, 61: 3}	https://doi.org/10.1371/journal.pone.0092031
GSE53598	36	Homo sapiens	Expression profiling by array	{}	{‘Male’: 36}	{‘60 years pre’: 5, ‘59 years post’: 2, ‘52 years pre’: 1, ‘57 years pre’: 3, ‘51 years post’: 1, ‘56 years post’: 1, ‘63 years post’: 2, ‘66 years post’: 1, ‘56 years pre’: 1, ‘51 years pre’: 1, ‘52 years post’: 1, ‘63 years pre’: 2, ‘59 years pre’: 2, ‘57 years post’: 3, ‘62 years post’: 2, ‘62 years pre’: 2, ‘66 years pre’: 1, ‘60 years post’: 5}	https://doi.org/10.1152/physiolgenomics.00174.2013
GSE58249	34	Homo sapiens	Expression profiling by array	{‘Vastus’: 34}	{}	{}	https://doi.org/10.1152/physiolgenomics.00024.2014
GSE59088	54	Homo sapiens	Expression profiling by array	{‘Vastus’: 54}	{‘Male’: 54}	{}	http://doi.org/10.1038/sdata.2014.41
GSE59363	42	Homo sapiens	Expression profiling by array	{}	{}	{65: 3, 48: 3, 51: 3, 53: 3, 54: 6, 57: 6, 59: 3, 60: 3, 61: 9, 62: 3}	https://doi.org/10.1007/s00125-015-3584-x
GSE60591	57	Homo sapiens	Expression profiling by high throughput sequencing	{‘Skeletal’: 125}	{‘Female’: 60, ‘male’: 65}	{}	https://doi.org/10.4161/15592294.2014.982445
GSE68072	23	Homo sapiens	Expression profiling by array	{‘Blood’: 23}	{}	{‘Young’: 23}	https://doi.org/10.1186/s12864-016-3388-5
GSE68585	20	Homo sapiens	Expression profiling by array	{‘Vastus’: 20}	{‘Male’: 16, ‘female’: 4}	{‘40 yrs’: 4, ‘48 yrs’: 4, ‘’45 yrs’: 4, ‘62 yrs’: 4, ‘66 yrs’: 4}	https://doi.org/10.1371/journal.pone.0160327
GSE71972	48	Homo sapiens	Expression profiling by high throughput sequencing	{‘Vastus’: 48}	{}	{}	https://doi.org/10.1113/JP272177
GSE72462	36	Homo sapiens	Expression profiling by array	{‘Muscle’: 36}	{‘Female’: 24, ‘male’: 12}	{64: 2, 56: 2, 37: 4, 39: 2, 40: 4, 42: 2, 43: 2, 45: 4, 48: 2, 54: 2, 24: 2, 58: 2, 28: 2, 61: 2, 62: 2}	https://doi.org/10.2337/db15-1723
GSE7286	24	Homo sapiens	Expression profiling by array	{}	{}	{}	https://doi.org/10.1152/physiolgenomics.00151.2006
GSE83352	84	Homo sapiens	Expression profiling by array	{‘Vastus’: 84}	{‘Male’: 38, ‘female’: 46}	{28: 2, 29: 4, 34: 4, 35: 2, 41: 2, 44: 4, 45: 4, 46: 2, 47: 2, 48: 4, 50: 2, 51: 6, 52: 4, 53: 4, 54: 8, 55: 6, 56: 4, 59: 4, 60: 2, 63: 8, 64: 2, 65: 4}	https://doi.org/10.1249/MSS.0000000000001041
GSE83578	64	Homo sapiens	Expression profiling by array	{}	{‘Female’: 16, ‘male’: 16}	{}	http://doi.org/10.1186/s12974-016-0758-5
GSE8479	65	Homo sapiens	Expression profiling by array	{}	{‘Female’: 34, ‘male’: 31}	{18: 2, 19: 3, 20: 5, 21: 6, 22: 6, 23: 2, 27: 1, 28: 1, 65: 5, 66: 4, 67: 5, 68: 4, 69: 4, 70: 2, 71: 2, 73: 6, 74: 2, 76: 1, 79: 2, 80: 1, 84: 1}	https://doi.org/10.1371/journal.pone.0000465
GSE87748	20	Homo sapiens	Expression profiling by high throughput sequencing	{‘Skeletal’: 20}	{}	{}	https://doi.org/10.3389/fendo.2016.00165
GSE9103	40	Homo sapiens	Expression profiling by array	{}	{}	{}	https://doi.org/10.2337/db08-0349
GSE9405	21	Homo sapiens	Expression profiling by array	{}	{}	{}	https://doi.org/10.1249/MSS.0b013e31818c6be9
GSE97084	103	Homo sapiens	Expression profiling by high throughput sequencing	{}	{}	{‘Young’: 57, ‘old’: 46}	https://doi.org/10.1016/j.cmet.2017.02.009
GSE99963	28	Homo sapiens	Expression profiling by high throughput sequencing	{‘Muscle’: 28}	{}	{}	https://doi.org/10.1038/s41598-017-15420-7

The included studies presented data on 7,080 individual samples, demonstrating a broad range in age distribution (1162 Young/<30, 1156 Middle age/30–60, and 262 Old/60<), gender composition (1739 Male 1236 Female), exercise types (4687 Aerobic/Endurance, 2162 Resistance/Strength, and 84 Immobilization), species (5150 Homo Sapiens/Humans, 955 Mus Musculus/Mice, and 975 Rattus Norvegicus/Rat), sequencing methods (3724 Microarray, 3345 Bulk-RNA-Seq, 10 SC-RNA-Seq) (Note that the subgroups were only counted if the dataset provided the information and therefore not all of the subgroups will add up to the 7080 total samples). Table 1 includes the sample breakdowns of these categories for each individual dataset.

To promote wider use of this database as a future resource, we’ve publicly released code (https://github.com/YoyoVosko/MacrophagesOntheRun) enabling the download of all the expression and annotation files from each of the 75 datasets directly from our database.

### 3.2 Exercise activates an immediate M1 pro-inflammatory macrophage surge

For each of the analyzed datasets, we processed each dataset such that all expression values were log2 normalized and boolean relationships were calculated between all pairs of genes in each dataset. Figure 1 illustrates the process through which the boolean relationships and network were created. Using the previously published boolean Macrophage polarization model from Ghosh et al., 2023 [1] we computed the composite macrophage score for every sample. A lower score indicated a higher expression of M1 Macrophage-associated genes, while a higher score indicated a greater presence of M2 Macrophage-associated genes. Two consistent and distinct patterns emerged across the dataset.

**Figure 3:**
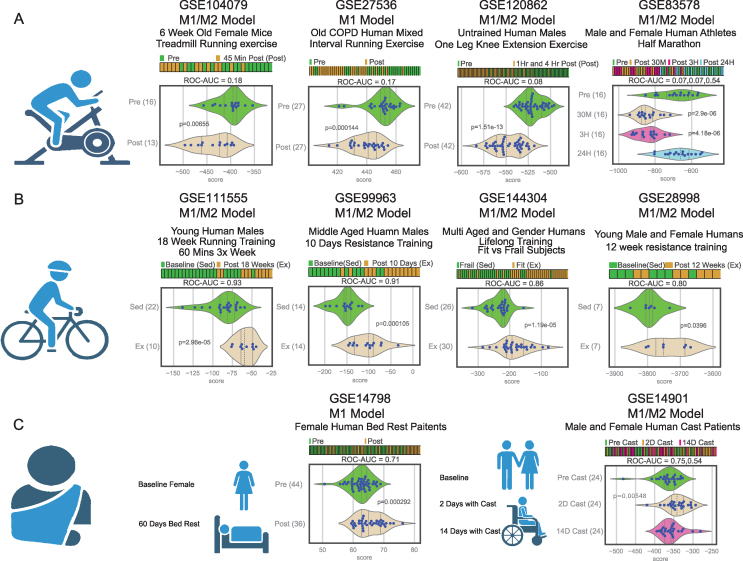
**Immediate exercise triggers the activation of M1 macrophages, while long term exercise yields the converse effect of M2 activation.** A) Immediate exercise results in a statistically significant reduction in “macrophage scores,” indicating the activation of M1 macrophages and an enhanced pro-immune response. This pattern is evident in selected datasets involving 6-week treadmill-trained mice (GSE104079 [52]), COPD human patients (GSE27536 [85]), male humans undergoing knee extension exercise (GSE120862 [65]), and male and female humans post–marathon (GSE83578 [21]). B) Prolonged exercise leads to an elevation in “macrophage scores,” indicative of the activation of M2 macrophages and a conducive environment for wound healing. Chosen datasets exhibiting this opposing pattern include young male human subjects engaged in aerobic training (GSE111555 [120]), resistance training alongside a high fat diet (GSE99963 [43]), lifelong exercise in both male and female young (20–30 yr) and old (75+ yr) subjects (GSE144304 [111]) and a 12 week resistance training regime for young male and female subjects (GSE28998 [34]). C) Immobilization studies illustrate a distinct contrast to the immediate exercise response, wherein higher post-exercise scores signify the effects of inactivity in direct opposition to those induced by exercise. The datasets presented encompass 60 days of bed rest in all female subjects (GSE14798 [27]) as well as observations at 2 and 14 days following quad brace in both male and female young patients (GSE14901 [20]). The scores for GSE27536 and GSE14798 were calculated with only the M1 associated genes.

The initial trend, observed across all datasets where samples were taken within 6 h of the last exercise session, regardless of previous exercise regimen, demonstrated a notable shift towards the prevalence of M1 macrophages. As depicted in Figure 3A, the samples collected post-exercise displayed a significantly lower “Macrophage Score” in comparison to those obtained prior to the exercise. This indicates an activation of M1 macrophages, leading to an amplified pro-inflammatory and pro-immune response that plays a crucial role in combating pathogens and other harmful diseases. These findings are consistent with the generally pro-inflammatory immediate effects of exercise [11].

It is noteworthy that this pattern is time-sensitive, with the M1 activation returning to the initial pre-exercise levels by 24-hours post-exercise. This aligns with the previously proposed notion that acute inflammation, when not prolonged into a chronic state, can hold substantial benefits for human health [109], a proposition supported by these findings.

### 3.3 Exercise sustain M2 reparative macrophages activation over time

The contrasting pattern, equally evident and opposite in nature, was discerned in all long-term exercise datasets. These data points were gathered during periods of rest, occurring 24 or more hours after the last exercise session following a multi-week training regimen. As depicted in Figure 3B, the post-exercise samples exhibited elevated scores in comparison to the pre-exercise samples, indicating an activation of M2 macrophages. This signifies a more proliferative environment, conducive to cellular repair, a process imperative for the restoration and rejuvenation of tissues subjected to tearing or damage during exercise. This pattern is consistently observed across both seasoned and novice participants [43], regardless of diverse sampling methods [31], various exercise conditions [90], and samples taken from both muscle and blood sources [32].

These results align with the well-documented anti-inflammatory effects of exercise [[16], [17], [18]]. The differing outcomes can thus be attributed to this transition between immediate and long-term exercise. Moreover, the equilibrium between the exercise induced M1 immune response and M2 wound healing process appears to hold the key to the beneficial aspects of exercise. Similar to how a higher heart rate during and immediately after exercise leads to a lower resting heart rate [110], the substantial surge in M1 macrophages immediately post-exercise allows for reduced levels of M1 macrophages during rest, consequently fostering higher levels of M2 activity.

### 3.4 Inactivity reverses effects of exercise on macrophages

Similar to heart rate, the intensity of M1/M2 activation escalates with increased levels of exercise. In total, 60% (45 out of 75) of all the datasets exhibited a statistically significant distinction between pre and post-exercise samples, indicating a substantial shift in activation for either M1 or M2 macrophages. This percentage rose to 75% (6 out of 8) when focusing solely on athletes (defined as subjects who had engaged in regular exercise prior to the study). This implies that the immune system and wound healing benefits of exercise are amplified with consistent physical activity. Conversely, it also underscores how sedentary individuals may already have heightened levels of inflammation, making them less responsive to immediate exercise effects. Nevertheless, these activations were consistently present in all samples, regardless of their statistical significance.

Figure 3C illustrates the inclusion of two immobilization studies. One study involved samples taken post days of bed rest [27], while the other looked at 2 and 14 days post quadriceps brace immobilization [20], in both cases minimal to no muscle movement occurred. In both cases, the post-immobilization samples exhibited statistically significant higher “macrophage scores,” indicative of an increase in M2 activity—an outright reversal of the immediate exercise response. These findings suggest that not only is the act of exercise itself advantageous to human health, but also that a lack of exercise and prolonged inactivity leads directly to a less active immune state. Similarly, the immediate exercise dataset in which both pre and post-exercise samples all yielded positive scores (corresponding to higher M2 levels) was Turan et al., 2011, which specifically examined the effects of exercise on Chronic Obstructive Pulmonary Disease (COPD) patients [85]. In these cases, the patient’s immune response (and correspondingly the M1 activation) appears less robust compared to the other studies, further emphasizing the similarity between the lack of exercise and a disease-related immune state.

### 3.5 Exercise Macrophage Activations patterns are consistent across all datasets

**Figure 4:**
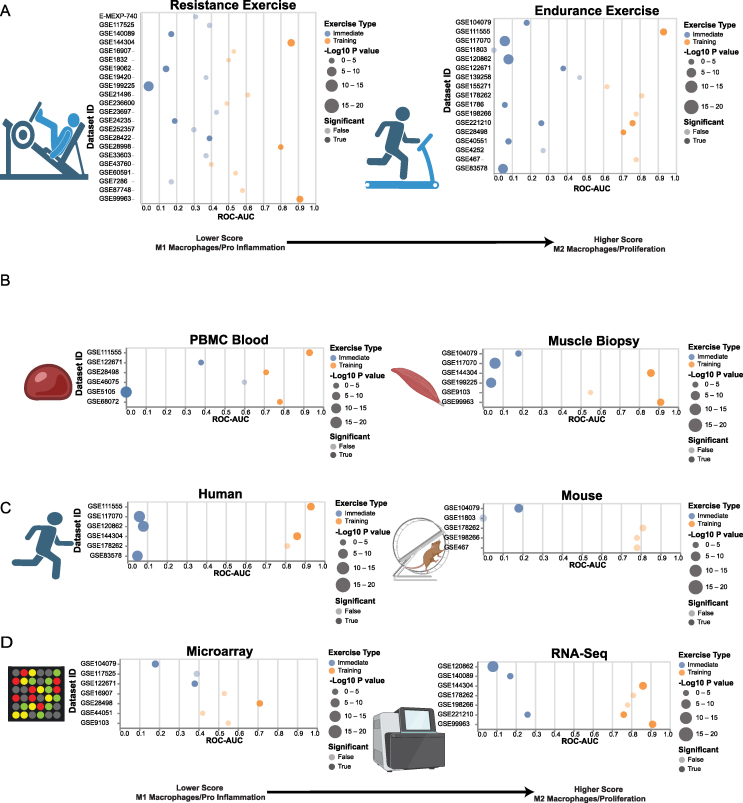
**Exercise Macrophage Activations are Consistent across diverse datasets.** A) Resistance and Endurance exercise, B) Blood and muscle biopsy sampling, C) Human and Mouse species, and D) RNA-Seq vs Microarray sequencing. In each section, dot plots depict exercise datasets on the y-axis, identified by their GSE accession numbers, while the x-axis represents the ROC-AUC scores obtained when attempting to differentiate pre and post-exercise data based solely on the composite “macrophage score”. In this context, a ROC-AUC value of 0 indicates that all post-exercise samples exhibit lower scores compared to pre-exercise samples, indicating a higher degree of M1 macrophage activation. Conversely, a ROC-AUC of 1 signifies that all post-exercise samples possess higher scores than pre-exercise samples, indicating a heightened level of M2 macrophage activation. The dot sizes are proportional to the -Log10(P Value) derived from a t-test comparing the pre and post-exercise groups, with statistically significant values displayed in solid shading, while non-significant datasets are slightly faded. Notably, all immediate exercise datasets exhibit ROC-AUC values below 0.5, aligning with M1 macrophage activation and an immune response. Conversely, regardless of dataset segmentation, all long term training exercise datasets showcase ROC-AUC scores surpassing 0.5.

As previously noted, the datasets aggregated for this meta-analysis exhibited considerable diversity in terms of included conditions and types of exercise. Despite this diversity, we consistently identified two distinct patterns of macrophage activation. Figure 4 illustrates the contrasting trends of immediate M1 and long term M2 activations. Regardless of how the datasets were segmented, whether by exercise type (Figure 4A), tissue sampling (Figure 4B), species (Figure 4C), or sequencing technology (Figure 4D), a consistent pattern emerged. Immediate post exercise datasets consistently displayed lower “macrophage scores,” indicative of the activation of M1 pro-immune response macrophages. Conversely, resting exercise datasets consistently exhibited higher scores, associated with the activation of M2 macrophages and the promotion of an anti-inflammatory, cell-repairing environment. The steadfastness of these findings suggests that while various exercises may have diverse effects on the human body, their impact on macrophages and the immune system at large is relatively straightforward and not subject to significant variation. This underscores the pivotal role of exercise in immune and wound healing responses, providing insight into why exercise has been observed to influence the immune system across a wide array of tissues [109]. Supplementary Table 1 includes the P-Values and ROC-AUC values differentiating between pre and post-exercise or sedentary and trained conditions for each of the 75 datasets within the database.

### 3.6 Gender, Intensity and Age Influence Macrophage Polarization in Humans

**Figure 5:**
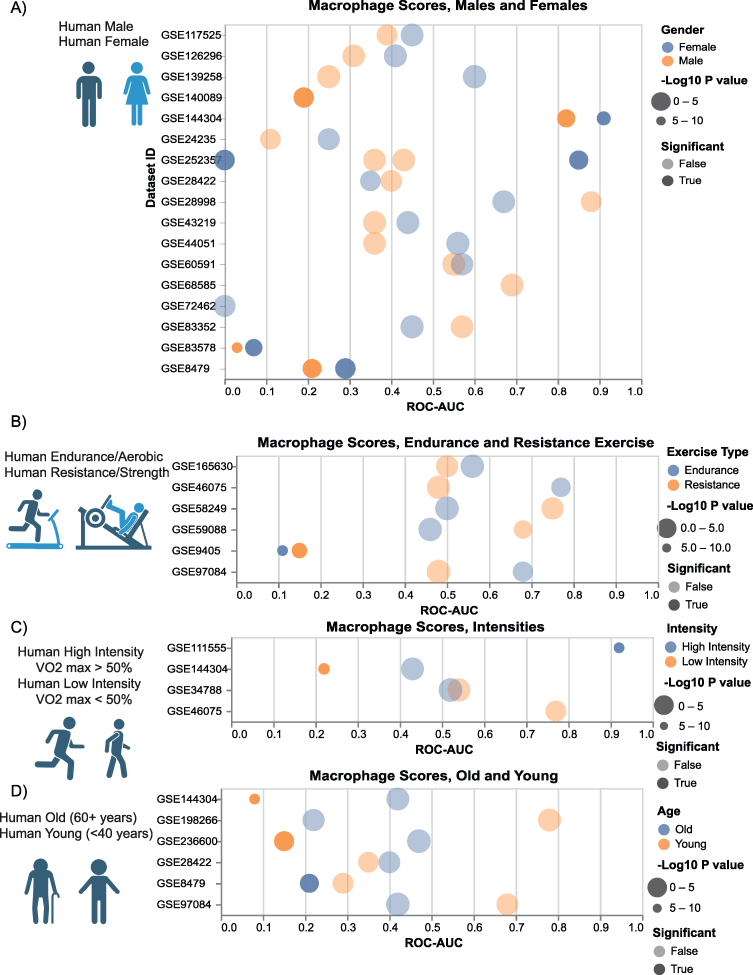
**Gender, Intensity and Age Influence Macrophage Polarization in Humans,** for datasets that included exercise data of multiple subgroups the pre/post or sedentary/trained macrophage scores were calculated for each group separately and compared on the same y-axis. A) Male and Female human subjects B) Endurance and Resistance exercise regimes C) Higher and lower intensity human subjects (based on VO2 max percentage) D) Younger and older human subjects. In each section, the dot plots depict exercise datasets on the y-axis, identified by their GSE accession numbers, while the x-axis represents the ROC-AUC scores obtained when attempting to differentiate pre/post or sedentary/trained exercise data based solely on the composite “macrophage score”. In this context, a ROC-AUC value of 0 indicates that all post-exercise samples exhibit lower scores compared to pre-exercise samples, indicating a higher degree of M1 macrophage activation. Conversely, a ROC-AUC of 1 signifies that all post-exercise samples possess higher scores than pre-exercise samples, indicating a heightened level of M2 macrophage activation. The dot sizes are proportional to the -Log10(P Value) derived from a t-test comparing the pre and post-exercise groups, with statistically significant values displayed in solid shading, while non-significant datasets are slightly faded. Notably exercise’s impact on M1 and M2 macrophage activation was stronger in female subjects than males, not noticeably different in endurance and resistance exercise types, stronger in higher intensity subjects, and stronger in younger subjects when compared to older ones.

To better understand how individual genders, exercise types, exercise intensities and ages contributed to the macrophage sample score, we conducted further analysis. For datasets that included exercise data on multiple subgroups, the pre/post or sedentary/trained macrophage scores were calculated for each group separately and compared to one another. The comparison of ROC-AUC and P values between the subgroups in the same datasets are shown in Figure 5A) Male and female human subjects B) Endurance and resistance human exercise regimes C) Higher and lower intensity human subjects, based on VO2 max percentage (Maximal Volume of Oxygen) D) Younger and older human subjects. While the immediate M1 and long term M2 patterns observed in Figure 3, Figure 4 remained consistent within all of these subgroups (wherein all the immediate datasets had a ROC-AUC value below 0.5 and all long term datasets a ROC-AUC value above 0.5) the specific contribution of different genders, exercise intensities and ages influenced the strength and significance of the macrophage polarization scores.

Our analysis of datasets including both male and female participants (Figure 5A) revealed a sex-specific effect on macrophage polarization. Female subjects generally exhibited stronger ROC-AUC scores, indicating a more pronounced shift towards M1 polarization (closer to 0) in immediate datasets and a more pronounced shift towards M2 polarization (closer to 1) in long-term training, compared to male subjects. Furthermore, female subjects demonstrated statistical significance in more datasets than the male subjects (7 vs. 5). This aligns with studies that have shown exercise to have sex specific differences, with women having a lower anti inflammatory response [21], alongside higher levels of cell regeneration [47] and cell growth [111], while men have been found to have more muscle growth and a stronger increase in mitochondria function when compared to women [24,47,111]. Our analysis suggests that the increased inflammation observed in women following exercise likely derives from a stronger immune response, as reflected by the higher M1 macrophage activity. It is possible that the stronger immune response in women results in greater tissue damage, leading to higher inflammation and a subsequent increase in cell regeneration. In contrast, the initially smaller immune response in men may have led to a correspondingly smaller repair response and in turn a weaker long term transition to M2 macrophage activity.

In datasets which included both resistance and endurance exercise groups (Figure 5B), we found no significant differences between the macrophage scores. Both types of exercise had similar effects on macrophage activity, with an equal number of significant results (1 each, on the same dataset [80]). Generally, resistance exercise has been shown to be more impactful on slow twitch fibers that lead to higher muscle growth [55,68]. While endurance exercise has a bigger impact on fast twitch muscles and in turn more metabolic related pathways [73,80,112]. Our datasets did not include the resolution of specific slow vs fast twitch fibers but suggests that both forms of exercise have a similar effect on both M1 and M2 macrophage activity.

In datasets which included different levels of exercise intensity (Figure 5C), we found that subjects that had engaged in higher-intensity exercise (VO2 max >50%) were associated with stronger macrophage polarization compared to those that had undergone lower-intensity exercise (VO2 max <50%). Higher intensity subjects also demonstrated more statistically significant results. This aligns with studies included in the analysis that have shown higher intensity exercise to have a larger effect on human gene expression [31,67]. Our analysis of these same studies highlights the activation of Macrophages as a significant aspect of these expression changes.

In datasets which included different human age groups (Figure 5D) younger individuals (<40 years) consistently had stronger macrophage scores than older subjects (60+ years). Similarly, the impact of exercise on younger subjects compared to their pre-exercise state was more consistently significant than in the older. Age has been shown to have a large impact on the effect of exercise, with old participants generally having a muted immune response to exercise [68,112,113]. This suggests that exercise can aid in reversing the effects of aging [23,35,55,68]. These results also suggest that the benefit exercise provides goes down with age, and that the older participants likely require higher intensity of exercise to achieve the same results as the younger age group. Supplementary Table 2 includes the P-Values and ROC-AUC values of the gender, exercise types, exercise intensity and age groups.

### 3.7 Identification of a new signature of muscle resident macrophage polarization

Given the significant impact of exercise on muscle, we aimed to refine the macrophage model to more accurately capture macrophage polarization within muscle tissues. To achieve this, we categorized the macrophage model genes into groups that most accurately differentiated exercise-induced changes in muscle samples and compared between these new potential gene models. Genes were taken from the original macrophage model [1], and evaluated based on their ability to differentiate between pre and post-exercise conditions in a muscle tissue exercise dataset [68]. Genes that showed a strong difference (absolute ROC-AUC value of at least 0.7) were selected and were divided into two groups based on their ROC-AUC values (Group 1 = >0.7, Group 2 = <0.3). These new gene groups included a significantly lower number of genes compared to the original set, with Group 1 containing 10 genes (2 M1, 8 M2) and Group 2 containing 25 genes (3 M1, 22 M2) in comparison to the original models 338 genes (48 M1, 290 M2).

**Figure 6:**
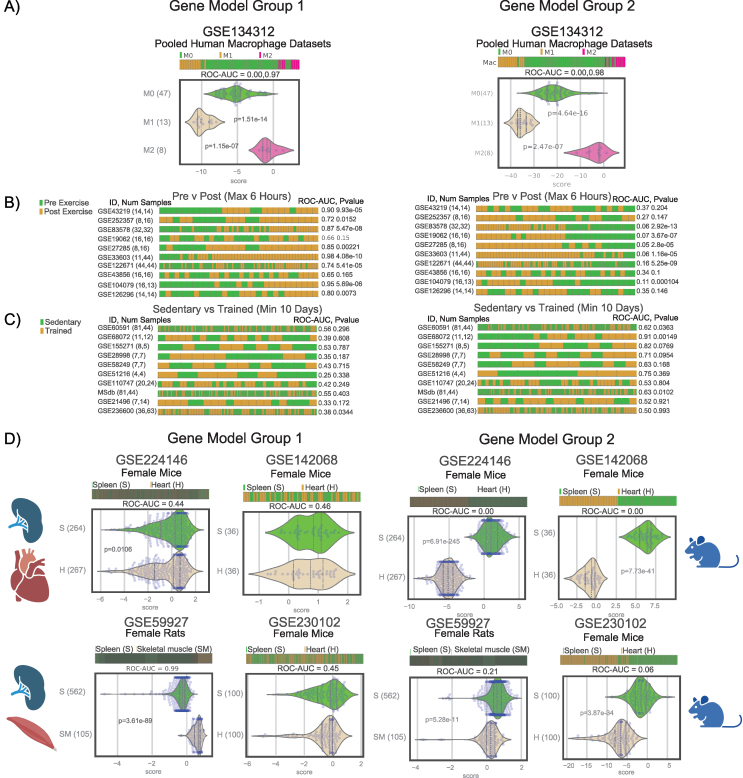
**Identification new Signature of Muscle Resident Macrophage Polarization.** A) Both Group 1 and Group 2 model gene sets are able to accurately separate and predict polarized macrophages. B) Pre and Post immediate exercise (within 6 h of exercise) C) Sedentary and Long term Trained (at least 10 weeks) individuals. Barplots show the Pre/Sendtary (in green) and Post/Exercised (in brown) group macrophage scores of both the Group 1 and 2 model. Higher Roc-Auc values represent a higher “M1” score while lower values represent a lower “M1” and higher M2 score. The two groups show opposite patterns to one another, with Group 1 having lower scores in the immediate exercise and higher scores in the long term training, and Group 2 having the exact opposite patterns. D) The expression of each of the gene model groups in both Spleen and Heart or Skeletal Muscle tissue. While the expression of Group 1 is not differentially distinct between the two tissue types, Group 2 shows a consistently higher expression in the macrophage rich spleen tissue as opposed to the muscle tissues identifying Group 2 as the Signature of Muscle Resident Macrophage Polarization.

As can be seen in Figure 6, the two gene set groups retain their ability to accurately predict and model the macrophage polarization itself when validated on a pooled dataset of 170 published macrophage samples (GSE134312) [101] (Figure 6A).

However, when tested on the 20 muscle tissue datasets from the assembled 75 database, the two groups show opposite patterns in both the immediate and long term exercise datasets. While Group 1 shows a higher macrophage score (more M2 macrophages) in the immediate and a lower score (more M1) in the long term, Group 2 is consistent with the overall macrophage model’s pattern of a lower score in the immediate and a higher score in the long term (Figure 6B,C). To better understand why Group 1 behaves differently, the gene groups were compared across spleen cells which are known to contain many macrophage cells [114], and heart/skeletal muscle tissues which contain relatively fewer macrophage cells [115].

By comparing the heart and skeletal muscle to the spleen in 3 non-exercise datasets (GSE142068, GSE230102, GSE59927) [[98], [99], [100]] and 1 exercise (GSE224146) dataset [106] the Group 1 model genes show no difference in expression between the two types of tissue, with expression signatures being equal in both tissue types (Figure 6D). Meanwhile the Group 2 model genes are differentiated between the tissue types, while maintaining higher macrophage signatures for the spleen tissues in multiple different datasets, aligning with the known higher macrophage content in the spleen (Figure 6D).

These results suggest that Group 2 is more reliable in predicting macrophage behavior across different tissues, especially when considering the varying macrophage content. Group 1 might be influenced by factors specific to muscle tissue and as such does not accurately model the increase in either M1 or M2 macrophages induced by exercise.

Group 2 identified a novel gene signature specific to muscle resident macrophage polarization, independent of muscle tissue gene expression. This 25-gene signature M1: APOL6, LIMK2, IL15; and M2: CLK2, CRTAP, DFFB, EEF1B2, MGAT4A, RPL14, RPL15, RPL17, RPL24, RPL35A, RPL9, RPS15A, RPS16, TTC31, ZNF266, MFNG, MKKS, NDUFB8, PAN2, SLC26A6, ZNF133, ZNF589, accurately models and predicts macrophage polarization within the context of muscle tissue and exercise. By isolating genes unaffected by muscle background expression, this signature provides a refined understanding of macrophage polarization dynamics in muscle. This signature is consistent with the original Macrophage Polarization Model in muscle tissues, while condensing the model to the core muscle macrophage genes.

## 4 Conclusions

Our study pioneers the first comprehensive meta-analysis of how exercise affects macrophage polarization. Through analysis of 75 diverse datasets using a Boolean-assisted Macrophage Polarization model, we uncover a compelling narrative: immediate exercise triggers activation of M1 pro-inflammatory macrophages, while the long-term effect orchestrates a shift towards M2 cell repair macrophages. This unveils a time-dependent manipulation of macrophage polarization by exercise, connecting the dynamic interplay between pro-immune response and cell repair pathways to the long-term health benefits reaped by exercise. Meanwhile, immobilization or reduced physical activity results in a reversal of the exercise-induced macrophage response, indicating the importance of consistent exercise for maintaining immune health. The observed macrophage activation patterns occurred reliably across different exercise types, tissues, species, and sequencing methods, suggesting a universal response to exercise. While the overall trend of M1 polarization in the short term and M2 polarization in the long term remained consistent across gender, exercise intensity and age, the specific contribution of these factors influenced the strength and significance of the polarization scores. With human females, higher intensity and younger subjects showing a stronger macrophage polarization response than male, lower intensity and older subjects. Our study also identified a novel Signature of Muscle Resident Macrophage Polarization gene set which accurately reflects the expression of macrophage polarization in muscle tissues using a small subset of genes that is separate from the gene expression of the muscle itself. This new signature of genes allows for the deeper and more accurate study of macrophage polarization within the muscle and allows for previous exercise and muscle datasets that had not previously studied macrophages to be used in this context, greatly increasing the number of applicable studies for this field.

Our findings are significant because macrophage polarization is linked to a variety of human diseases, including cancer, cardiovascular disease, and autoimmune disorders [116]. It suggests that an imbalance between pro-immune and cell repair responses could pave the way for disease development while the balance between the M1 and M2 macrophages is essential for optimal health. For example, excessive M1 activity can lead to cardiovascular problems [117], while dominant M2 polarization could foster a more aggressive tumor environment and impede treatment responsiveness [118,119]. Ultimately, exercise serves as an immunomodulator profoundly impacting the immune system by influencing macrophage polarization.

Crucially, our study highlights the importance of a dynamic transition state between M1 and M2 macrophages for optimal health. This knowledge paves the way for developing personalized exercise programs tailored to patients with specific health conditions. Overall, our study sheds light on exercise’s crucial role in maintaining human health through the modulation of macrophage polarization. Our findings provide valuable insights into the molecular state of healthy individuals and the pivotal role of a balanced immune system in promoting human well-being.

## Lead contact

Further information and requests for resources and reagents should be directed to and will be fulfilled by the lead contact Debashis Sahoo (dsahoo@ucsd.edu) and Yotam Voskoboynik (yvoskoboynik@ucsd.edu).

## CRediT authorship contribution statement

**Yotam Voskoboynik:** Writing – review & editing, Writing – original draft, Visualization, Validation, Software, Resources, Methodology, Investigation, Formal analysis, Data curation, Conceptualization. **Andrew D. McCulloch:** Writing – review & editing, Supervision, Project administration, Funding acquisition. **Debashis Sahoo:** Writing – review & editing, Validation, Supervision, Software, Resources, Project administration, Methodology, Investigation, Funding acquisition, Formal analysis, Data curation, Conceptualization.

## Materials availability

This study did not generate new unique reagents.

## Declaration of competing interest

None.

## Data Availability

All datasets are publicly available on GEO, TableS1 details all studies in this analysis. All original code has been made public and deposited at: https://github.com/YoyoVosko/MacrophagesOntheRun

## Appendix A Supplementary data

Multimedia component 1 Supplementary Table 1: **Macrophage Dataset Sample Scores,** provides a comprehensive overview of the statistical analysis performed on macrophage datasets. It includes P-values and ROC-AUC scores for comparing Pre vs Post or Sedentary vs Exercised sample groups. Additionally, the table specifies whether the statistical test refers to immediate or long-term training effects, reports the -Log10 P values for each comparison, and indicates significant differences between groups (P ≤ 0.05). Notably, some datasets with both immediate and long-term timescale groupings are represented multiple times in the table to facilitate detailed comparison. Multimedia component 1

Multimedia component 2 Supplementary Table 2: **Macrophage Subgroup Sample Scores,** provides a comprehensive overview of the statistical analysis performed on the gender, exercise type, exercise intensity and age subgroup comparisons. It includes P-values and ROC-AUC scores for comparing Pre vs Post or Sedentary vs Exercised sample groups, as well as the Gender, Exercise Type, Exercise Intensity and Age subgroup for applicable datasets, datasets which were not part of each comparisons have that columns left blank. Additionally, the table reports the -Log10 P values for each comparison and indicates significant differences between groups (P ≤ 0.05). Multimedia component 2
